# Perceived Stress, Stigma, Traumatic Stress Levels and Coping Responses amongst Residents in Training across Multiple Specialties during COVID-19 Pandemic—A Longitudinal Study

**DOI:** 10.3390/ijerph17186572

**Published:** 2020-09-09

**Authors:** Qian Hui Chew, Faith Li-Ann Chia, Wee Khoon Ng, Wan Cheong Ivan Lee, Pei Lin Lynnette Tan, Chen Seong Wong, Ser Hon Puah, Vishalkumar G. Shelat, Ee-Jin Darren Seah, Cheong Wei Terence Huey, Eng Joo Phua, Kang Sim

**Affiliations:** 1 Department of Research, Institute of Mental Health, Singapore 539747, Singapore; qian_hui_chew@imh.com.sg; 2 National Healthcare Group Residency, Singapore 138543, Singapore; faith_chia@ttsh.com.sg (F.L.-A.C.); phua.eng.joo@ktph.com.sg (E.J.P.); 3 Department of Rheumatology, Allergy and Immunology, Tan Tock Seng Hospital, Singapore 308433, Singapore; 4 National Healthcare Group Internal Medicine Residency Program, Singapore 138543, Singapore; wee_khoon_ng@ttsh.com.sg; 5 Department of Gastroenterology and Hepatology, Tan Tock Seng Hospital, Singapore 308433, Singapore; 6 National Healthcare Group Education Office, Singapore 138543, Singapore; ivan_wc_lee@nhg.com.sg; 7 Department of Psychiatry, Tan Tock Seng Hospital, Singapore 308433, Singapore; lynnette_tan@ttsh.com.sg; 8 National Healthcare Group Infectious Diseases Residency Program, Singapore 138543, Singapore; chen_seong_wong@ttsh.com.sg; 9 Department of Infectious Diseases, Tan Tock Seng Hospital, Singapore 308433, Singapore; 10 National Healthcare Group Respiratory Medicine Residency Program, Singapore 138543, Singapore; ser_hon_puah@ttsh.com.sg; 11 Department of Respiratory and Critical Care Medicine, Tan Tock Seng Hospital, Singapore 308433, Singapore; 12 National Healthcare Group Post-Graduate Year 1 Training Program, Singapore 138543, Singapore; vishal_g_shelat@ttsh.com.sg; 13 Department of General Surgery, Tan Tock Seng Hospital, Singapore 308433, Singapore; cheong_wei_huey@ttsh.com.sg; 14 National Healthcare Group Family Medicine Residency Program, Singapore 138543, Singapore; darren_ej_seah@nhgp.com.sg; 15 National Healthcare Group Polyclinics, Singapore 138543, Singapore; 16 National Healthcare Group General Surgery Residency Program, Singapore 138543, Singapore; 17 Department of General Medicine, Khoo Teck Puat Hospital, Singapore 768828, Singapore; 18 National Healthcare Group National Psychiatry Residency Program, Singapore 138543, Singapore; 19 West Region, Institute of Mental Health, Singapore 539747, Singapore

**Keywords:** COVID-19, residency, psychological responses, healthcare workers

## Abstract

This study aimed to explore changes in psychological responses (perceived stress, traumatic stress, stigma, coping) over time in residents, as well as their predictors. The level of perceived stress, traumatic stress, stigma, and coping responses were assessed using the Perceived Stress Scale, Impact of Event-Revised, Healthcare Workers Stigma Scale, and Brief Coping Orientation to Problems Experienced (COPE) Inventory, respectively. We collected responses from 274 residents at baseline and 221 residents at 3 months follow-up (timepoint 2) from the National Healthcare Group (NHG) residency programs in Singapore. All residents reported lower perceived stress and lower perceived stigma compared to baseline. Use of avoidance coping was associated with all three psychological responses (perceived stress, traumatic stress, and stigma) across the two timepoints. Compared to baseline, specific factors associated with perceived stress and traumatic stress at timepoint 2 were living alone, less problem solving, and seeking social support. Residency programs should encourage active coping strategies (e.g., seeking social support, positive thinking, problem solving) among residents, and proactively identify residents who may be at higher risk of psychological sequelae due to circumstances that contribute to isolation.

## 1. Introduction

The first case of Coronavirus Disease 2019 (COVID-19) in Singapore was recorded on 23 January 2020. Since the start of 7 February 2020 until now, the local government has recognized the disease as severe and easily transmissible from person to person and has undertaken strict infection control measures to manage the outbreak [1]. Healthcare professionals, including residents, were deployed to high-risk areas such as the National Centre for Infectious Diseases (NCID) where patients who test positive or are suspected to have COVID-19 with respiratory symptoms are being sent for treatment [2]. In our recent review of extant studies examining COVID-19-related psychological sequelae [3], we found that healthcare workers (HCWs) reported relatively high rates of anxiety (33.0%), traumatic stress (14.6%), and stigmatization (32.6% [4]).

These psychological responses to the pandemic could change over time depending on the severity and rapidity of the spread of the disease, among other factors. Close monitoring of these responses is needed in order for us to provide adequate and appropriate support to frontline HCWs. There is also a need to clarify whether the associated predictors of psychological responses have changed over time, so that psychological support can be better optimized for HCWs. To the best of our knowledge, there is no published study that examines the COVID-19-related psychological sequelae over time among frontline HCWs, and especially within residents.

Our previous study found that residents who were deployed to high-risk areas (NCID) had lower perceived stress levels [5]. Higher perceived stigma level was associated with higher levels of perceived stress and post-traumatic symptoms, while avoidance as a coping strategy was associated with higher levels of perceived stress, traumatic stress symptoms, and perceived stigma [5]. This current paper aims to examine how the earlier psychological responses within residents in training across multiple specialties have evolved over the course of the pandemic, as well as the relevant associated demographic factors and coping strategies.

## 2. Experimental Section

All residents from the National Healthcare Group (NHG) Residency Programs in Singapore were invited to participate in this survey. This included residents from 27 specialties, of which 20 residency programs are U.S. Accreditation Council for Graduate Medical Education-International (ACGME-I)-accredited, and seven are accredited by the Joint Committee on Specialty Training (JCST), Singapore. These specialties were grouped under two broad categories, medical (medical specialties, radiology, family medicine, psychiatry) and surgical disciplines (surgery, anesthesia, emergency medicine). Similar to the survey done at the first timepoint [5], we sent out emails with a brief study description and a link to the survey hosted on Qualtrics for a period of approximately 3.5 weeks from 8th June 2020 to 2nd July 2020 (timepoint 2). This was approximately three months after the first timepoint of the survey conducted from 5th March 2020 to 10th April 2020 at baseline. Participation was voluntary and the survey was anonymized. It was approved as an exempt study under the National Healthcare Group’s Institutional Review Board, Singapore (NHG DSRB Ref: 2020/00220). Patients or the public were not involved in the design, or conduct, or reporting, or dissemination plans of our research.

Residents were administered a structured questionnaire comprising four main outcome rating scales and a section on sociodemographic details including gender, age, marital status, living arrangements, job experience, deployment to high-risk areas (NCID), and deployment outside of one’s usual job scope.

The 10-item Perceived Stress Scale (PSS) [6] was used to measure levels of perceived stress in residents. It is rated on a 5-point Likert scale from 0 (Never) to 4 (Very Often), with higher total score reflecting a higher level of perceived stress.

The 22-item Impact of Event Scale-Revised (IES-R) [7] was used to measure the level of traumatic stress. It is rated on a 5-point Likert scale ranging from 0 (Never) to 4 (Very Often), with a higher score indicating a higher level of traumatic stress. It comprises three subscales (Intrusion, Avoidance, and Hyperarousal). The mean scores of each of these three subscales and the total score were used in our analyses.

The adapted 12-item Healthcare Workers Stigma Scale (HWSS) [8,9] was used to assess residents’ level of perceived stigma. It was rated on a 4-point Likert scale ranging from 1 (Strongly Disagree) to 4 (Strongly Agree). Items can be grouped into four subscales: Personalized stigma, Disclosure concerns, Concerns about public attitudes, and Negative self-image [8]. Personalized stigma relates to the respondent’s own perceived consequences, personal experiences, or fears of rejection when others find out that the respondent is a HCW. Disclosure concerns measures the extent to which respondents control information or keep their occupation as an HCW a secret from others. Concerns about public attitudes refers to what most people in general think about HCWs, and their possible responses when they come to know about the respondents’ occupation as a HCW. Negative self-image measures the extent to which respondents feel inferior to others as a result of their occupation as an HCW [8]. A higher score reflects a higher level of perceived stigma.

The 28-item Brief Coping Orientation to Problems Experienced (COPE) questionnaire [10] was used to assess the type and frequency of coping strategies employed by residents. It is rated on a 4-point Likert scale ranging from 1 (Never) to 4 (Very Often). The items can be grouped into four main coping responses: seeking social support, problem solving, positive thinking, and avoidance [11]. A higher score indicates more frequent use of a specific coping response.

All four outcome measures have been widely used in infectious disease outbreak studies to evaluate the psychological morbidity in HCWs within different healthcare settings [12,13,14].

We used the Statistical Package for Social Sciences (SPSS) Version 23 (IBM Corp., Armonk, NY, USA) to compare results obtained from both timepoints with independent t-tests for continuous variables and chi-square for categorical variables. Results were adjusted for multiple comparisons using the Benjamini–Hochberg method wherever appropriate. We also employed linear regression analyses to examine the predictors of PSS, IES-R, and HWSS at each timepoint, while controlling for gender, seniority, marital status, living arrangement, exposure to patients with respiratory symptoms, and deployment to high-risk areas (NCID). Direction Dependence Analysis (DDA [15]) was then used to further clarify the direction of relationship between the predictors and outcome variables. DDA allows us to evaluate the theory of one-directional relationships between variables (e.g., x → y) as compared to other directional alternatives (e.g., y → x) using skewness and kurtosis of the data [15]. The theory (x → y) is supported when the distribution of variable y is closer to normality than x, the residual distribution of x → y is closer to normality than that of y → x, and the independence assumption of residuals and predictor holds for x → y while at the same time being violated for y → x [15]. A *p*-value of <0.05 (2-tailed) was taken to indicate statistical significance.

## 3. Results

### 3.1. For all Residents

Overall, 274 residents (response rate of 49.2%) participated at baseline (timepoint 1) and 221 (response rate 39.7%) participated at follow-up (timepoint 2). The sociodemographic features of respondents, and a comparison of differences between responses obtained at the first and second timepoints, are presented in Table 1. The proportion of residents deployed to NCID, their levels of perceived stress, and perceived stigma were significantly different from timepoint 1. A greater proportion of residents had been, or were currently deployed to NCID. Their level of perceived stress and stigma was significantly lower than at timepoint 1, particularly for concerns pertaining to disclosure of their identity as HCWs, and concerns about public attitudes towards HCWs.

### 3.2. For Residents Deployed Outside Job Scope

At baseline, 88 residents (32.1%) were deployed outside their usual job scope. In the past few weeks prior to the timepoint 2 survey, 71 residents (32.1%) were deployed outside their usual job scope. Residents reported less perceived stigma as compared to the previous timepoint, particularly in the area of concerns about public attitudes towards HCWs, but this was not significant after adjustment for multiple comparisons (see Table 2).

### 3.3. Predictors of Psychological Responses

Perceived stress was positively predicted by the use of avoidance as a coping strategy and levels of perceived stigma, and was negatively predicted by the use of positive thinking for responses obtained from both timepoints. Residents not deployed to high-risk areas also experienced more perceived stress than those who did. Of note, within responses obtained from timepoint 2, we found that living alone predicted increased stress (see Table 3). The data met the assumptions of normality of residuals, linearity, homoscedasticity, and absence of multicollinearity.

Traumatic stress as measured by the IES-R was positively predicted by use of avoidance as a coping strategy, and levels of perceived stigma across both timepoints. Compared with baseline, responses from timepoint 2 indicated that use of problem solving and use of social support as coping strategies were negative and positive predictors of traumatic stress, respectively (see Table 3). In order to further clarify the relationship between social support and traumatic stress, we employed DDA [15]. We hypothesized that greater traumatic stress would lead to more frequent use of social support seeking as a coping mechanism (IES-R €→ Social support), while controlling for gender, seniority, living arrangements, marital status, exposure to patients with respiratory symptoms, and deployment to high-risk areas (NCID). Both IES-R total and COPE social support scores met conditions of non-normality as evaluated by the Shapiro–Wilk test (*p* < 0.001). First, we evaluated the direction of the effect, and results indicated that skewness and excess-kurtosis values were close to zero for COPE social support scores, while IES-R scores significantly deviated from normality in terms of skewness and excess-kurtosis (both *p* < 0.001). This provides evidence in line with the hypothesis of IES-R → Social support. Next, we evaluated the residuals, which showed that the residuals for the hypothesized model IES-R − Social support were significantly closer to normality than those of the alternative model Social support − IES-R (both *p* < 0.001). Lastly, we analyzed the independence properties of the models. Results showed that the independence assumption held true for the hypothesized model (IES-R → Social support) while at the same time was violated for the alternative model (Social support → IES-R) (both *p* < 0.05). Hence, we are able to conclude that, taking into account the covariates, greater traumatic stress was likely to lead to more social support seeking.

Perceived stigma was positively associated with use of avoidance as a coping strategy and traumatic stress across both timepoints (see Table 3). Responses from timepoint 2 did not indicate any other predictors of perceived stigma that were not found in timepoint 1. A summary of the main findings across both timepoints are presented in Table 4.

## 4. Discussion

This longitudinal study on residents during the ongoing COVID-19 pandemic revealed three main findings. First, residents reported less perceived stress and stigma over time. Second, use of avoidance coping was positively associated with all three psychological responses (perceived stress, traumatic stress, and stigma) across the two timepoints. Third, compared to baseline, specific factors that were negative predictors of perceived stress and traumatic stress at timepoint 2 were living with others, use of problem solving, and seeking social support.

Overall, residents reported lower stress levels at the second timepoint compared to baseline. This could be attributed to several reasons. First, all residents were faced with an unprecedented situation at the height of the pandemic during the first timepoint, but have managed to gradually adapt to the situation over time. Local healthcare institutions have ensured a constant supply of Personal Protective Equipment (PPE) for all residents, and thus far, no infections of HCWs were directly attributable to patient contact. No deaths have been reported among HCWs as well. These factors served to assure residents of their safety at work even during the pandemic. Second, healthcare institutions and residency programs would have had the chance to fine-tune their communication process and procedures over time. This reduces the sense of uncertainty and the need for residents to adapt to rapidly changing policies and protocols. Most of the residents surveyed at the second timepoint would have been deployed to high-risk areas, with some being deployed several times. The familiarity with the situation reduces any anticipatory anxiety that may be present at the first timepoint of the study. In addition, residents were given the opportunity to lead and contribute to the writing of protocols, conferring a sense of ownership and control over the situation. Third, the number of infectious cases sent to healthcare facilities (acute hospitals/intensive care units) has gone down due to the expansion of community facilities, resulting in less strain on the healthcare system. Due to sufficient manpower allocation, residents deployed to high-risk areas and healthcare facilities for COVID-19 duties are often on 8-h shifts. For residents in some specializations with longer working hours on a regular day, these rotational deployments have provided some respite, which could have reduced perceived stress.

Residents also reported less perceived stigma at the second timepoint relative to the baseline. This could be a result of concerted nationwide efforts including within the healthcare institutions to raise awareness of HCWs’ contributions during this pandemic. The support of members of the community towards HCWs was also publicized on various media platforms, leading to less stigmatization over time [16]. In particular, residents had fewer concerns about disclosing their profession as a HCW, and about the attitude of the public towards HCWs. Residency programs have also taken active steps to recognize the efforts of residents through awards and features in newsletters. As a result of initiatives to recognize the efforts of HCWs, positive narratives could have been created over time, both within the professional medical community as well as the general public, that allow residents to maintain a shared view of their contribution as beneficial and essential to society [17]. In addition, residents deployed outside their job scope reported less perceived stigma at the second timepoint, with fewer concerns about the attitude of the public towards HCWs. This could be due to the support, for example, through thank-you cards, food and drinks given by the general public particularly towards HCWs in high-risk areas (NCID), which have demonstrated in tangible terms that public attitudes are largely positive.

Predictors of stress that were common across both timepoints included the use of avoidance as a coping strategy (positive predictor), use of positive thinking (negative predictor), perceived stigma (positive predictor), and being deployed to high-risk areas (NCID) (negative predictor). In addition, we found that living alone was a positive predictor of stress in the second timepoint of the survey. Residents living alone could be facing issues of isolation and lack of social connections and support. Given that this predictor was only significant at the second timepoint, it could also suggest that the loss of social support occurred over time due to external circumstances (e.g., social distancing measures, increased workload during the pandemic), or that its effect on stress is less immediate as compared with the other factors explored in our study. Stricter social distancing measures locally were also only enforced towards the end of our baseline assessment, and were only gradually lifted near the start of our second timepoint. This could have contributed to the loss of social support seen over time as well. The stress-buffer hypothesis suggests that identifying and checking in more frequently with residents in training who live alone may help buffer the negative effects of stress on mental health and well-being [18], and that consistent follow-ups with our learners should be conducted throughout the course of the pandemic.

Predictors of traumatic stress that were common across both timepoints include use of avoidance as a coping strategy (positive predictor), and perceived stigma (positive predictor). In addition, we found that use of problem solving and social support were negative predictors of traumatic stress at the second timepoint. The tendency to employ problem solving as a coping strategy can be conceptualized as both a trait and a state. As a trait, some residents may not favor the use of problem solving as a coping strategy, which could have contributed to greater traumatic stress when faced with unprecedented events such as the pandemic. As a state, residents currently experiencing traumatic stress may also rely less on problem solving as a coping strategy, preferring alternatives such as seeking social support, as found in this study [19]. This could also be a function of the nature of the stressor, as situations that are appraised as being solvable are more likely to induce problem-focused coping, while situations that are appraised as being unchangeable are more likely to induce emotion-focused coping [20]. The current COVID-19 pandemic is characterized by its high transmissibility, infectivity, as well as the current absence of vaccine at the time of writing [21]. This could have led to a sense of helplessness and a preference for emotion-focused coping amongst residents. Residency programs can further support residents during this challenging period by providing avenues where they can seek social support (e.g., setting up peer-support groups, making psychological counseling available), give regular updates on the situation in order to reduce feelings of uncertainty, and provide feedback on how residents’ efforts have contributed positively to the fight against COVID-19.

Predictors of perceived stigma common across both timepoints included use of avoidance as a coping strategy (positive predictor), and traumatic stress (positive predictor). Use of avoidance coping was positively associated with all three psychological responses (stress, traumatic stress, and stigma) across timepoints. Examples of avoidance coping as measured by the Brief COPE Inventory included behavioral disengagement (“give up trying to deal with it”, “give up attempt to cope”), self-blame (“criticizing myself”, “blame myself for things that happen”), denial (“refusing to believe it has happened”), and distraction (“doing something to think about it less”). These measures capture attempts to avoid the situation through both external (outward behavior) and internal (mental processes) strategies. A recent longitudinal study seeking to clarify the directionality of relationship between avoidance coping and trauma found that avoidance coping was more common in individuals with a history of traumatic stress [22]. In addition, they found that when social support seeking was higher, this predicted a decrease in subsequent trauma exposure. The authors suggested that social support seeking could represent a more proactive coping style, which allows them to gather resources that protect against exposure to, and/or the negative effects of traumatic stress [22]. This could serve to explain why residents experiencing traumatic stress related to the pandemic were more likely to employ social support seeking as a coping strategy.

### 4.1. Practical Points

Our results suggest four areas of focus for residency programs particularly during this pandemic. First, program and faculty should seek to raise awareness of common psychological responses amongst residents and actively identify those at higher risk of experiencing negative psychological sequelae such as increased perceived stress, traumatic stress, and stigma. Second, residency programs should seek to encourage active coping through problem solving and seeking social support. This could be more helpful in reducing negative psychological responses than merely discouraging avoidance coping [22]. We also found that less frequent use of positive thinking to cope was a common predictor of perceived stress across both timepoints, and is another form of active coping that should be encouraged in residents. This includes accepting the reality of the situation and looking for something good in what is happening [10]. Third, faculty members may try to actively identify residents who are facing circumstances that require them to be isolated (such as those residents staying alone, under self-imposed quarantine, on sick leave) in order to check-in on them regularly. Residency programs could also form peer support groups to encourage residents to maintain a sense of connectedness even in the midst of social distancing measures, through phone/video calls, text messages, and various social media platforms. Fourth, residency programs should continue to build up and encourage positive narratives surrounding the work and contributions of residents during the pandemic. This serves as a buffer against perceived or actual stigma that residents may experience from others around them [17].

### 4.2. Limitations

In order to preserve the anonymity of responses and to encourage greater participation amongst residents, we were unable to match the responses of residents who took part in the survey at both timepoints. Participation in the second timepoint of the study was not a mandatory requirement for participation in the first timepoint of the study and vice-versa so as to allow us to collect responses from residents who may not necessarily be available at both timepoints. We were also keen to get an accurate picture of the situation on the ground in order to better support our residents. Hence, greater care was taken to collect only necessary demographic information and to avoid assigning codes to residents which could have allowed us to link responses from residents who participated at both timepoints. In addition, we did not examine other factors such as intercurrent stressors (e.g., life events) or personality characteristics, which can also affect the psychological responses shared within the study. Nonetheless, this study provides an overview of the psychological responses and changes over time observed in residents in the short term and may be helpful for residency programs in pre-empting their needs and designing interventions to better support residents.

## 5. Conclusions

In conclusion, we found that residents reported experiencing less stress and stigma over time despite the ongoing pandemic. This could be attributed to better workflow and processes that have been fine-tuned over time, gradual adaptation to the situation, as well as tangible support towards HCWs demonstrated by the public. Avoidance coping was a significant predictor of adverse psychological responses across both timepoints. Of note, residents may also be experiencing more social isolation during the pandemic, which could further increase their risk of negative mental health outcomes. Residency programs should encourage the use of active coping strategies amongst residents (seeking social support, positive thinking, problem solving), and take active steps to identify and better support residents who may be at higher risk of psychological sequelae due to circumstances that contribute to isolation such as those staying alone or on self-quarantine.

## Figures and Tables

**Table 1:** Demographic and clinical variables of all residents (baseline vs. follow-up).

	Baseline (n = 274)	Follow-Up (n = 221)	Between-Group Differences	
			Test Statistic	*p* Value
Age, years (Mean, S.D)	30.6 (2.67)	30.8 (2.87)	*t* = −0.941	0.347
Sex (n, %)			Χ^2^ = 0.139	0.709
Male	133 (48.5)	111 (50.2)		
Female	141 (51.5)	110 (49.8)		
Marital status (n, %)			Χ^2^ = 0.000023	0.996
Single	145 (52.5)	117 (52.7)		
Married	129 (46.7)	104 (46.8)		
Living Arrangement (n, %)			Χ^2^ = 0.004	0.949
Alone	24 (8.76)	19 (8.60)		
With others	250 (91.2)	202 (91.4)		
Discipline			Χ^2^ = 2.352	0.125
Medical disciplines	191 (69.7)	167 (75.6)		
Surgical disciplines	83 (30.3)	53 (24.0)		
Duration of work as HCW, years (Mean, S.D)	6.07 (2.55)	6.12 (2.61)	*t* = −0.215	0.830
Exposed to patients with respiratory symptoms (n, %)	222 (81.0)	169 (76.5)	Χ^2^ = 1.527	0.217
Deployed to areas outside usual job scope (n, %)	88 (32.1)	71 (32.1)	Χ^2^ = 0.000006	0.998
Deployed to NCID (n, %)	81 (29.6)	110 (49.8)	Χ^2^ = 21.089	<.001
PSS scores (Mean, S.D)	28.1 (6.63)	27.0 (5.87)	*t* = 2.084	0.038
IES-R total scores (Mean, S.D)	15.0 (14.6)	14.5 (14.4)	*t* = 0.404	0.686
IES-R Intrusion subscale (Mean, S.D)	0.723 (0.738)	0.673 (0.660)	*t* = 0.765	0.445
IES-R Avoidance subscale (Mean, S.D)	0.695 (0.703)	0.704 (0.694)	*t* = −0.133	0.894
IES-R Hyperarousal subscale (Mean, S.D)	0.609 (0.700)	0.573 (0.732)	*t* = 0.539	0.590
HWSS total score (Mean, S.D)	22.5 (6.84)	20.8 (6.92)	*t* = 2.618	0.009
HWSS Personalized Stigma subscale (Mean, S.D)	5.09 (1.73)	4.81 (1.72)	*t* = 1.684	0.093
HWSS Disclosure Concerns subscale (Mean, S.D)	6.27 (2.29)	5.80 (2.37)	*t* = 2.150	0.032
HWSS Concerns about Public Attitudes subscale (Mean, S.D)	6.61 (2.28)	5.95 (2.26)	*t* = 3.081	0.002
HWSS Negative Self-image subscale (Mean, S.D)	4.51 (1.90)	4.21 (1.71)	*t* = 1.718	0.086
COPE Social Support subscale (Mean, S.D)	2.01 (0.572)	2.08 (0.598)	*t* = −1.356	0.176
COPE Problem Solving subscale (Mean, S.D)	2.24 (0.661)	2.23 (0.635)	*t* = 0.296	0.767
COPE Avoidance subscale (Mean, S.D)	1.46 (0.427)	1.48 (0.409)	*t* = −0.458	0.647
COPE Positive Thinking subscale (Mean, S.D)	2.40 (0.588)	2.38 (0.629)	*t* = 0.461	0.645

Abbreviations: COPE = Brief COPE Inventory; HCW = Healthcare worker; HWSS = Healthcare Workers’ Stigma Scale; IES-R = Impact of Event Scale-Revised; NCID = National Center for Infectious Diseases; PSS = Perceived Stress Scale; S.D. = Standard Deviation.

**Table 2:** Demographic and clinical variables of residents deployed outside usual job scope (baseline vs. follow-up).

	Baseline (n = 88)	Follow-up (n = 71)	Between-Group Differences		
			Test Statistic	*p* Value	Adjusted *p* Value
Age, years (Mean, S.D)	30.2 (2.79)	30.5 (2.38)	*t* = −0.667	0.505	0.694
Sex (n, %)			Χ^2^ = 2.512	0.113	0.355
Male	52 (59.1)	33 (46.5)			
Female	36 (40.9)	38 (53.5)			
Marital status (n, %)			Χ^2^ = 0.090	0.765	0.842
Single	45 (51.1)	38 (53.5)			
Married	43 (48.9)	33 (46.5)			
Living Arrangement (n, %)			Χ^2^ = 0.145	0.703	0.814
Alone	9 (10.2)	6 (8.45)			
With others	79 (89.8)	65 (9.15)			
Discipline			Χ^2^ = 10.510	0.001	0.022
Medical disciplines	43 (48.9)	52 (74.3)			
Surgical disciplines	45 (51.1)	18 (25.7)			
Duration of work as HCW, years (Mean, S.D)	5.97 (2.82)	5.87 (2.27)	*t* = 0.224	0.823	0.862
Exposed to patients with respiratory symptoms (n, %)	78 (88.6)	65 (91.5)	Χ^2^ = 0.368	0.544	0.704
Deployed to NCID (n, %)	61 (69.3)	59 (83.1)	Χ^2^ = 4.031	0.045	0.248
PSS scores (Mean, S.D)	27.5 (7.56)	26.2 (6.04)	*t* = 1.183	0.239	0.478
IES-R total scores (Mean, S.D)	17.4 (18.0)	14.1 (15.0)	*t* = 1.189	0.237	0.521
IES-R Intrusion subscale (Mean, S.D)	0.845 (0.897)	0.660 (0.710)	*t* = 1.376	0.171	0.418
IES-R Avoidance subscale (Mean, S.D)	0.783 (0.823)	0.677 (0.662)	*t* = 0.856	0.394	0.578
IES-R Hyperarousal subscale (Mean, S.D)	0.721 (0.905)	0.564 (0.829)	*t* = 1.093	0.276	0.467
HWSS total score (Mean, S.D)	24.1 (7.73)	21.3 (7.68)	*t* = 2.194	0.030	0.220
HWSS Personalized Stigma subscale (Mean, S.D)	5.49 (1.98)	4.92 (1.93)	*t* = 1.719	0.088	0.387
HWSS Disclosure Concerns subscale (Mean, S.D)	6.69 (2.42)	6.06 (2.57)	*t* = 1.517	0.132	0.363
HWSS Concerns about Public Attitudes subscale (Mean, S.D)	7.09 (2.42)	6.05 (2.34)	*t* = 2.622	0.010	0.110
HWSS Negative Self-image subscale (Mean, S.D)	4.85 (2.32)	4.26 (1.90)	*t* = 1.644	0.102	0.374
COPE Social Support subscale (Mean, S.D)	2.07 (0.642)	2.12 (0.561)	*t* = -0.543	0.588	0.719
COPE Problem Solving subscale (Mean, S.D)	2.33 (0.771)	2.21 (0.581)	*t* = 1.064	0.289	0.454
COPE Avoidance subscale (Mean, S.D)	1.58 (0.561)	1.48 (0.440)	*t* = 1.099	0.273	0.501
COPE Positive Thinking subscale (Mean, S.D)	2.49 (0.615)	2.47 (0.626)	*t* = 0.193	0.848	0.848

Abbreviations: COPE = Brief COPE Inventory; HCW = Healthcare worker; HWSS = Healthcare Workers’ Stigma Scale; IES-R = Impact of Event Scale-Revised; NCID = National Center for Infectious Diseases; PSS = Perceived Stress Scale; S.D = Standard Deviation.

**Table 3:** Risk Factors for Mental Health Outcomes Amongst Residents in Training from National Healthcare Group Residencies at the 2nd timepoint.

Variable	Multivariate Analysis			
	B	β	95% CI for B	*p* Value
PSS, stress				
Females (vs. males)	0.960	0.082	−0.352–2.273	0.151
Seniors (vs. juniors)	0.485	0.040	−0.869–1.838	0.481
Married (vs. single)	0.370	0.031	−0.984–1.723	0.590
Living with others (vs. alone)	−3.537	−0.167	−5.863–(−1.211)	0.003
Exposed to patients with respiratory illness	0.054	0.004	−1.546–1.653	0.947
Deployed to NCID	−1.912	−0.163	−3.281–(-0.543)	0.006
COPE Social Support	1.594	0.162	−0.002–3.190	0.050
COPE Problem Solving	−0.716	−0.077	−2.268–0.837	0.364
COPE Avoidance	6.911	0.480	4.829–8.994	<0.001
COPE Positive Thinking	−1.829	−0.195	−3.250–(−0.409)	0.012
HWSS total score	0.134	0.157	0.024–0.244	0.017
IES-R, PTS symptoms				
Females (vs. males)	−1.043	0.036	−3.860–1.774	0.466
Seniors (vs. juniors)	−1.288	−0.043	−4.194–1.618	0.383
Married (vs. single)	0.284	0.010	−2.622–3.189	0.847
Living with others (vs. alone)	−0.826	−0.016	−5.819–4.166	0.744
Exposed to patients with respiratory illness	0.536	0.016	−2.897–3.969	0.759
Deployed to NCID	−00.810	−0.028	−3.749–2.129	0.587
COPE Social Support	4.481	0.186	1.056–7.906	0.011
COPE Problem Solving	−4.418	−0.194	−7.751–(−1.086)	0.010
COPE Avoidance	21.463	0.609	16.994–25.933	<0.001
COPE Positive Thinking	−2.239	−0.098	−5.288–0.810	0.149
HWSS total score	0.477	0.228	0.241–0.714	<0.001
HWSS, Stigma				
Females (vs. males)	0.500	0.036	−1.153–2.153	0.551
Seniors (vs. juniors)	1.016	0.071	−0.676–2.708	0.238
Married (vs. single)	−1.504	−0.109	−3.180–0.172	0.078
Living with others (vs. alone)	1.725	0.070	−1.240–4.690	0.253
Exposed to patients with respiratory illness	0.451	0.028	−1.544–2.446	0.656
Deployed to NCID	1.443	0.105	−0.287–3.174	0.102
COPE Social Support	−0.746	−0.065	−2.775–1.282	0.469
COPE Problem Solving	−0.807	−0.074	−2.775–1.161	0.420
COPE Avoidance	5.447	0.324	2.335–8.560	0.001
COPE Positive Thinking	0.193	0.018	−1.610–1.996	0.833
PSS total score	0.090	0.077	−0.103–0.282	0.358
IES-R total score	0.145	0.303	0.057–0.232	0.001

Abbreviations: B = Unstandardized Beta; β = Standardized Beta; CI = Confidence Interval; COPE = Brief COPE Inventory; HWSS = Healthcare Workers’ Stigma Scale; IES-R = Impact of Event Scale-Revised; NCID = National Center for Infectious Diseases; PSS = Perceived Stress Scale; PTS = Post-traumatic stress.

**Table 4:** Summary of findings across both timepoints.

Psychological Responses							
		Perceived Stress		Traumatic Stress		Stigma	
		T_1_	T_2_	T_1_	T_2_	T_1_	T_2_
Perceived stress		–	↓ (over time)	–	–	↑	↑
Traumatic stress		–	–	–	–	↑	↑
Stigma		↑	↑	↑	↑	–	↓ (over time)
Deployed to NCID		↓	↓	–	–	–	–
Living alone		–	↑	–	–	–	–
Brief COPE Inventory	Avoidance coping	↑	↑	↑	↑	↑	↑
	Problem solving	–	–	–	↓	–	–
	Seeking social support	–	–	–	↑	–	–
	Positive thinking	↓	↓	–	–	–	–

Abbreviations: T_1_ = Timepoint 1; T_2_ = Timepoint 2.
